# Reducing the energy cost of running using a lightweight, low-profile elastic exosuit

**DOI:** 10.1186/s12984-021-00928-x

**Published:** 2021-08-30

**Authors:** Jaeha Yang, Junil Park, Jihun Kim, Sungjin Park, Giuk Lee

**Affiliations:** School of Mechanical Engineering, Chung-Ang University, 06974 Seoul, South Korea

**Keywords:** Passive exosuit, Running assistance, Assistive device, Metabolic cost, Spatiotemporal parameters

## Abstract

**Background:**

Human beings can enhance their distance running performance with the help of assistive devices. Although several such devices are available, they are heavy and bulky, which limits their use in everyday activities. In this study, we developed a lightweight running assistive device with a low-profile design. The device applies a flexion moment to the hip according to the hip extension within a specific range of motion to assist running.

**Methods:**

A passive exosuit was fabricated using textile materials and elastic bands. The deformation of the suit was measured and compensated for in the design. The fabricated suit was tested on eight participants (age: 24.4 ± 3.8 y; height: 1.72 ± 0.05 m; weight: 74.5 ± 6.1 kg) who were instructed to run on a treadmill at a speed of 2.5 m/s. Through indirect calorimetry, the metabolic rate was measured for the no-suit condition and three band conditions. Variations in the spatiotemporal parameters were measured using a motion capture system and force-sensing resistors (FSRs).

**Results:**

When using the fabricated device, seven out of the eight participants exhibited a reduced metabolic rate in at least one of the three band conditions. An average reduction of − 4.7 ± 1.4% (mean ± standard error of the mean (s.e.m.), two-sided paired t-test, p = 0.017) was achieved when using the best-fitting bands compared to the average of the two no-suit conditions. No statistically significant changes were observed in the spatiotemporal parameters, except for the stance duration in the medium assistance force condition.

**Conclusions:**

The proposed passive exosuit, which has a low weight of 609 g and small extrusion of 2.5 cm from the body in standing posture, can reduce the metabolic rate during running. The proposed device can potentially be used every day owing to its low-profile design and low weight, thereby overcoming the limitations of existing portable devices targeting the hip joints.

**Supplementary Information:**

The online version contains supplementary material available at 10.1186/s12984-021-00928-x.

## Background

Human running performance has the potential to be improved through the use of enhancing devices. Studies have shown that the metabolic energy cost is linearly related to the time consumed in running a certain distance [1, 2]. Based on these studies, many researchers have attempted to develop assistive devices that can reduce the metabolic cost during running, and a few of them have succeeded in achieving this goal [3].

Based on their assistive strategies, devices that can reduce the metabolic cost while running can be classified into active and passive devices, and the former has been studied extensively [4–7]. Devices of this type use external energy sources such as batteries to exert assistive forces on specific parts of the body, thereby reducing the biological moment needed to perform the target motion. Kim et al. developed an exosuit that used batteries to actively support hip extension [4]; tests on nine participants running at a speed of 2.5 m/s showed that a metabolic cost reduction of 4% could be achieved when running with the proposed device compared to running without it. However, the use of powered support posed certain limitations. Notably, the device had a relatively high weight of 5.0 kg owing to the use of batteries and motors. Moreover, the exosuit was bulky and extruded from the back of the waist, which rendered it cumbersome in daily life.

To overcome the inevitable drawbacks associated with the high weight and bulkiness of active devices, researchers have focused on passive devices based on simple unpowered mechanisms. In particular, passive devices do not require any external energy sources to function, thereby eliminating the need for heavy and bulky components such as batteries and motors. Passive devices provide support through an external mechanical element, such as a spring, to replace the negative work during the deceleration phase that is originally the user’s burden. For example, if a spring that deforms with the motion requiring deceleration is introduced, the deformation of the spring can perform a portion of the negative work from the motion, reducing the human energy consumed for the target motion.

Based on this principle, Nasiri et al. proposed a passive device [8] that incorporated a leaf spring as the elastic component, which generated torsional force while twisting. The leaf spring was composed of sheet metal and had a rectangular cross-section. In general, before the maximum extension or flexion of the thigh is attained during running, a person exerts a moment in the direction opposite to that of the thigh movement to decelerate, which is negative work. In the proposed device, both thighs were connected by using the leaf spring, and thus, the negative work from both thighs could be reduced while storing energy in the spring. The stored energy was released during reacceleration of the thighs, thereby reducing the positive work. Tests conducted with 10 participants showed that the metabolic cost incurred when running at a speed of 2.5 m/s with the proposed device was 8% lower than that incurred while running without it. Through the use of the passive mechanism, the device weight was significantly decreased to 1.8 kg. However, the suit was still heavy and bulky compared to everyday wear owing to the weight and extrusion of the leaf spring.

Simpson et al. also proposed a lighter and less bulky passive device compared to those previously reported for running [9]. The authors noted that connecting the legs by using an elastic band increased the energy optimal stride frequency during running, which resulted in a metabolic cost reduction of 6.4% while running at 2.7 m/s. The weight of the device was approximately 50 g, extremely low for a wearable device, which could be attributed to the use of only a single elastic band attached to the dorsal surface of each shoe. Although the possibility of outdoor use of this device was demonstrated through pilot tests, problems arising from the bands getting caught *in* terrain obstacles of daily life due to both the ankles being connected by the elastic band remain to be addressed.

Considering these aspects, in this study, we developed a lightweight low-profile passive device that can enhance the running economy of the user. To overcome the size and weight issues of the existing passive devices, the device was designed as a type of exosuit fabricated with textiles used in apparel design and rubber bands to decrease the overall weight and extrusion from the body. The hip joint generates a flexion moment near the maximum extension of the thigh as the thigh decelerates and reaccelerates. During this range of motion, the magnitude of the generated flexion moment increases almost linearly with the extension of the hip. Considering this biological feature, the exosuit was designed to passively exert flexion moments on the hip joint during thigh movement in the range of motion through elastic bands placed in front of each thigh. Moreover, the exosuit was designed so that the passively exerted moments increase according to the extension of the thigh by the increased resilience of the elastic band.

The human hip joint exhibits a passive elastic joint moment–angle relationship due to the stretching of the soft tissues [10]. In addition to the muscle-generated moments, the human hip joint passively acts as a flexion moment during extension. From mid-stance to mid-swing, the psoas tendon functions like a spring that stretches with extension, absorbing energy, later releasing the energy at toe-off. This passive energy storage and release helps conserve energy, thereby rendering the human running gait more energy efficient [11]. Because the proposed suit adds an external spring to the body, which serves as the psoas tendon during running, it can likely enhance the energy saving during running, leading to a higher running efficiency.

When the elastic bands are elongated, they deliver a force to the suit that causes the suit to deform. To consider this deformation of the suit while designing the bands, suit stiffness models were experimentally evaluated. Considering the individual differences in the optimal assistance levels of support devices, three bands with different stiffness levels were designed for testing. To verify the assistive performance of the fabricated exosuit, tests were performed with eight participants running at a speed of 2.5 m/s. Indirect calorimetry was used to calculate the differences in the metabolic energy cost for each condition. Notably, wearing a wearable device can alter the natural gait of the wearer, and this aspect should be analyzed to perform a comprehensive evaluation of wearable device performance. For example, Simpson et al. showed that spatiotemporal changes occurred when running with the exotendon, which led to a difference in the metabolic cost [9]. Therefore, a motion capture system and force-sensing resistors (FSRs) were used to analyze the effects of the exosuit on the spatiotemporal parameters of running. The force exerted by the exosuit was measured using a loadcell and synchronized with data from the motion capture system.

## Exosuit design

### Assist region

**Fig. 1:**
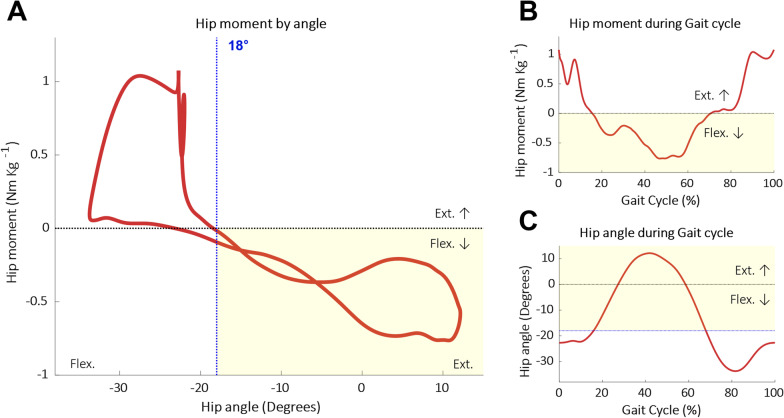
Hip moment and angle data obtained during running at 2.5 m/s. The target assist region, in which the flexion moment increases with the hip extension, is marked in yellow. Averaged data from seven participants, reported by Kim et al., were used. **A** Hip moment according to hip angle during running. A moderately linear correlation can be seen in the yellow region. **B** Changes in hip moment according to GC. (C) Changes in hip angle according to GC

To determine the appropriate target assist region, biological hip moment data were analyzed. Running motion data from seven participants running at 2.5 m/s, provided by Kim et al. as supplementary material [4], were used for the analysis. The average hip moments according to the hip angles, along with the average hip moment and angle during the running gait, are shown in Fig. 1. Based on the biological hip moment data, the hip joint is required to generate flexion moments in the range of motion between a flexion angle of 18° and maximum extension; this region is marked as yellow in Fig. 1. Moreover, the flexion moment in this region has a moderately linear correlation with the extension of the hip angle (R^2^ = 0.53 for the region with the hip flexion moment).

Considering this biological feature, the exosuit was designed to passively exert flexion moments on the hip joint by using the elastic bands placed in front of each thigh while the hip joint moved in this range of motion. The elastic band was designed to be in a slack state at hip angles between a flexion angle of 18° and maximum flexion to ensure that the band did not exert any moment on the hip. When the hip is inclined at an angle of 18° in the flexion direction, the band starts to elongate and generates a passive assistive moment on the hip joint. Moreover, the passively exerted moment was designed to increase as the thigh moved toward maximum extension with the increase in the resilience of the elastic band due to its elongation.

### Suit design

**Fig. 2:**
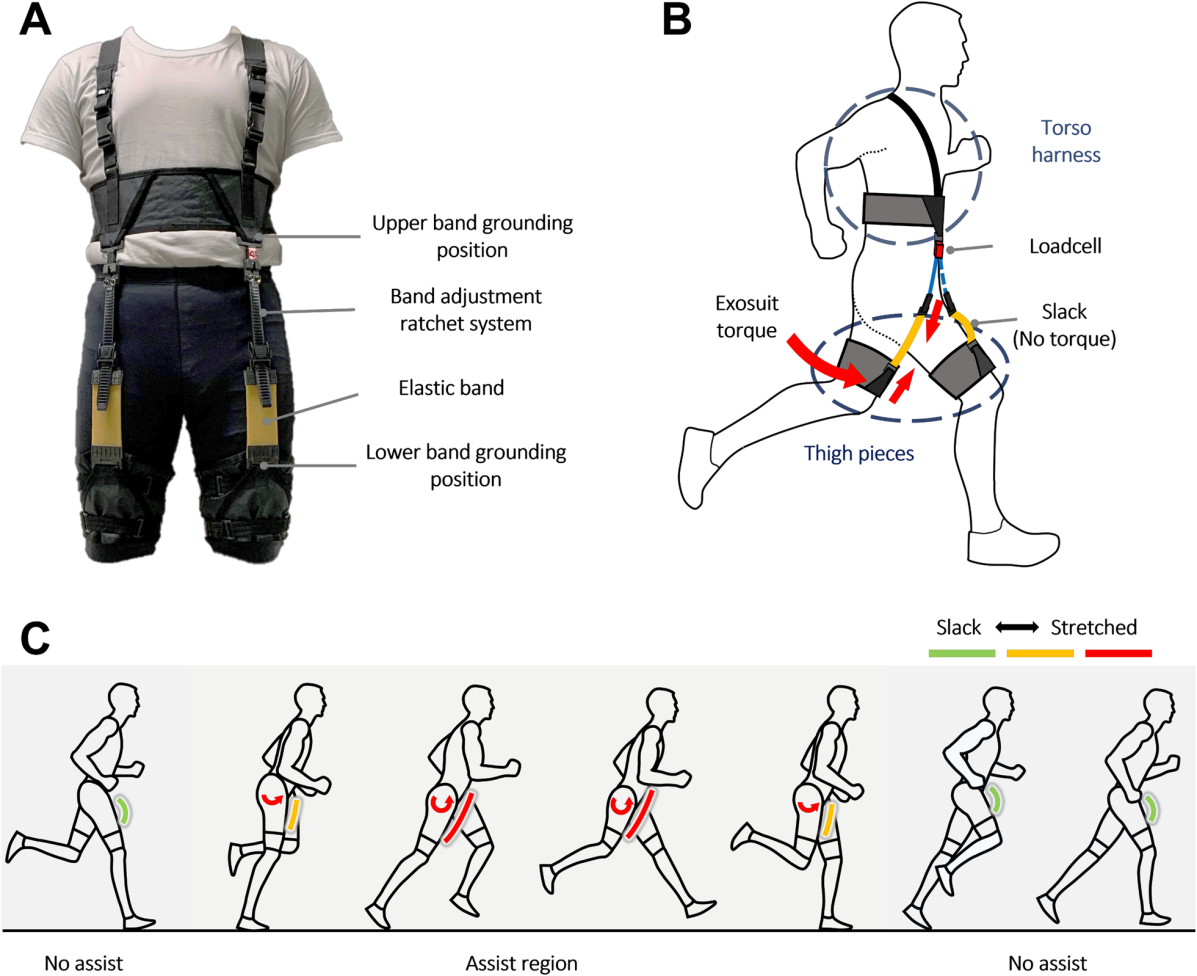
Design and working principle of passive exosuit. **A** Design of passive exosuit. The device consists of a torso harness for upper grounding and two thigh pieces for lower grounding. The device has several buckles that can be adjusted to fit the body shape of the user. **B** Working principle of passive exosuit. Extension of the hip leads to energy storage in the band, which is released during the flexion phase of the hip. **C** Working schematic of passive exosuit. The band stretches as the thigh moves to extension, exerting a flexion moment on the hip joint

An exosuit capable of assisting hip flexion was fabricated using textiles and elastic bands, as shown in Fig. 2. To minimize the suit deformation from the force of the band, a composite fabric-based textile (CT5K.18/wov4, Dyneema, USA) was used as the base material owing to its high toughness and low weight. Another layer of thin polyester-nylon-based textile was placed on the sides facing the body to reduce slippage between the exosuit and clothes worn under it. Tight-fitting sweatpants made of polyester were required to be worn underneath the thigh pieces to enhance the tactile sensation and prevent slippage. The “Exoband” fabricated by Panizzolo et al., which could provide hip flexion moments for the elderly and facilitate walking, was referenced in the design of the proposed suit [12].

The exosuit consisted of five parts: two thigh pieces, one torso harness with shoulder straps, and two band modules that could be attached between the torso harness and each thigh piece in the upper and lower band grounding positions, as depicted in Fig. 2A. Because the body sizes of users may vary considerably, the length of the band modules was designed to be adjustable using a ratchet system to enable customization. Furthermore, the shoulder straps and radius of the torso harness and the radii of the thigh pieces were designed to be adjustable to ensure that the suit can fit different body shapes. The loadcell was attached to the band module to measure the forces from the band exerted on the exosuit during running. The fabricated exosuit weighed 609 g, and the specific weights of the device components are presented in the supplementary material.

### Suit deformation analysis

Owing to the forces exerted on the suit by the band, the suit undergoes deformation that is nonlinear compared to the exerted force. This deformation may result in less elongation of the band than intended if not considered in advance. To estimate the effects of the band on the hip joint in the design process, this deformation must be considered. Therefore, a simulator that could estimate the assistance profile of a band according to the hip angle was developed.

**Fig. 3:**
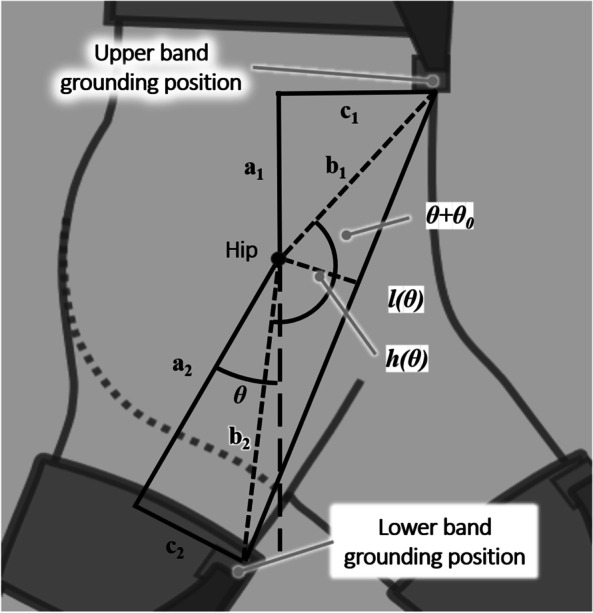
Mathematical model of distance between upper and lower grounding positions of exosuit, according to hip angle

Kinematic modeling of the human thigh with the flexion assistance force in the sagittal plane of the leg (Fig. 3), as suggested by Wei et al. [13], was performed to calculate the displacement between the upper and lower grounding positions on the body according to the hip angles. Parameters a1 and a2 were set as 0.15 m and 0.3 m, respectively, and c1 and c2 were set as 0.12 m and 0.08 m, respectively, based on the size of the human body and upper and lower band grounding positions on the body. Through a1, a2, c1, c2, and $\theta$, the initial length of the band module, ${l}_{0}$, and length of the band module at hip angle $\theta$, ${l}_{\theta }$, could be calculated using trigonometric principles. The displacement ${\delta }_{total}(\theta )$ was expressed as in Eq. (1).

1 $$\delta_{total} \left( \theta \right) = l_{\theta } - l_{0}$$

For the proposed exosuit, ${\delta }_{total}$ involved two parts, as expressed in Eq. (2), namely, elongation of the band and suit deformation.

2 $$\delta_{suit} + e_{band} = \delta_{total}$$

where ${{\varvec{\delta}}}_{{\varvec{s}}{\varvec{u}}{\varvec{i}}{\varvec{t}}}$ is the displacement from the deformation of the suit and ${{\varvec{e}}}_{{\varvec{b}}{\varvec{a}}{\varvec{n}}{\varvec{d}}}$ is the elongation of the elastic band. This relationship was required in the design process of the band modules to provide the appropriate assistance. The following section presents the derivation of the two terms.

### Suit deformation model

**Fig. 4:**
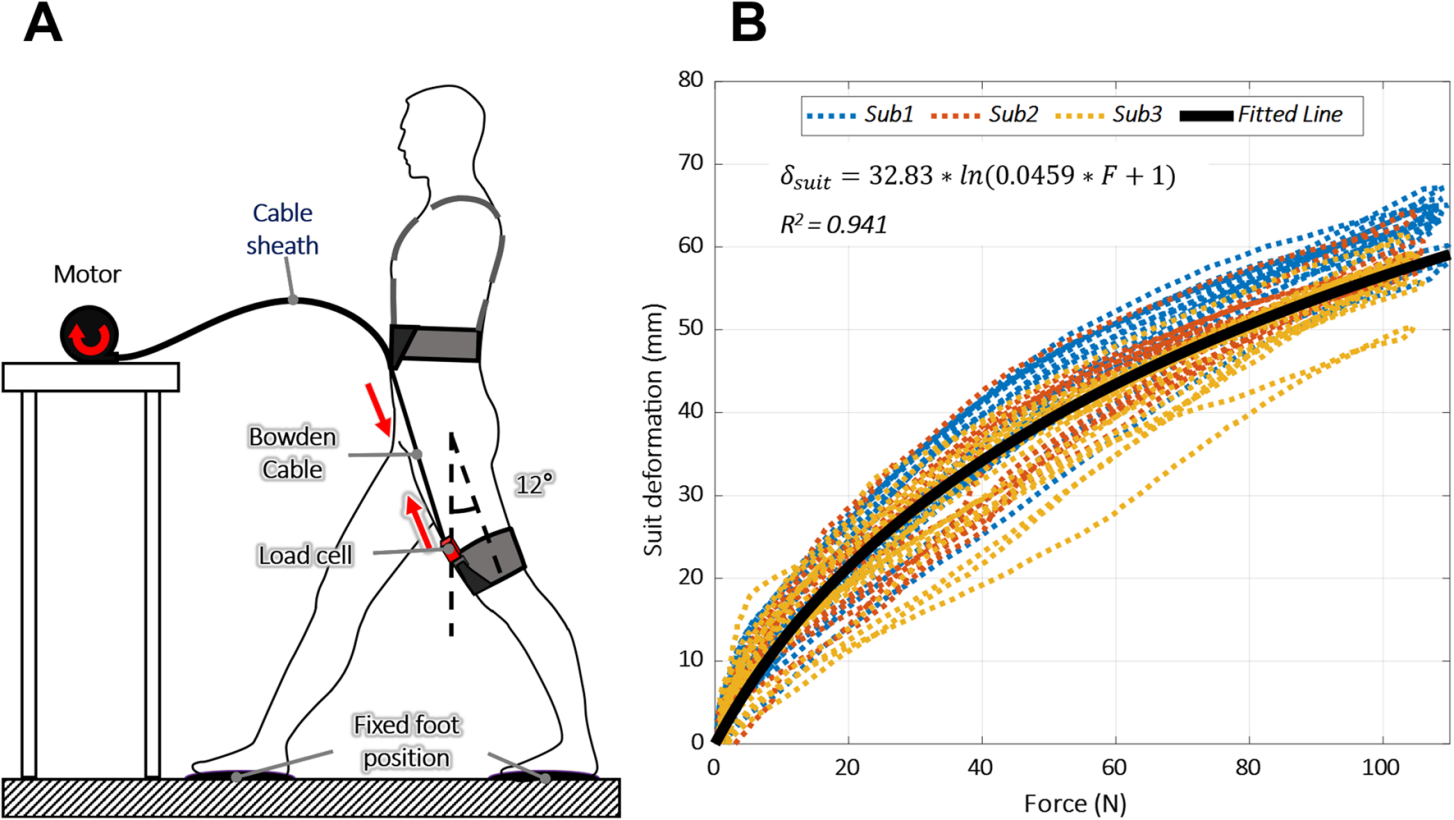
Suit deformation analysis. **A** Test setup for formulating suit deformation model based on force from band. **B** Suit deformation analysis test results. The colored dotted lines indicate the original test data from the three participants, and the solid black line is the fitted curve

To compensate for the suit deformation, we formulated the suit stiffness model by using the measurement methods suggested by Lee et al. [14]. Specifically, we used a BLDC motor (EC-4 pole 30-305015, Maxon Motor, Switzerland) with a gearhead (GP 32 HP-326664, Maxon Motor) to pull a Bowden cable (FCP-04DB, RESPONSE, Taiwan, used with cable housing BHL100, Jagwire, Taiwan) connecting the upper band grounding position of the torso harness and lower band grounding position of the thigh pieces receiving the force from the band module, as shown in Fig. 4. The received force was directly measured from the cable by using the loadcell. The deformation of the suit was estimated to be the displacement of the cable position from its initial position, caused by pulling from the motor. The cable position at which the loadcell data changed from zero to a positive value was set as the initial position of the suit before deformation. In the test, it was assumed that the participant was completely still and that no extension occurred in the length of the Bowden cable.

To acquire the data for deriving the suit stiffness model, we tested the device on three male participants (age: 24.8 ± 0.9 y; height: 1.72 ± 0.08 m; weight: 75.3 ± 6.8 kg) while they stood in a posture with a hip extension of 12°, which is similar to a 48% gait cycle (GC) in running. We selected this posture because the exosuit was designed to exert maximum force on the wearer at this point of the GC. The received force and deformation of the suit were recorded from each participant while the cable was pulled at a constant rate of 0.05 m/s until the peak force reached 100 N. We repeated the tests 10 times and used all the recorded data to derive the stiffness model.

The suit stiffness model was derived by fitting the recorded data to Eq. (3) proposed by Lee et al. [14].

3 $$\delta_{suit} = C_{s1} \ln (C_{s2} F + 1)$$

where ${\delta }_{suit}$ is the deformation of the suit and Cs1 and Cs2 are the model coefficients. The original test data and fitted curve using Eq. (3) are shown in Fig. 4.

### Band selection for three-level assistance

According to previous studies, the optimal assistance level varies among individual participants when using supportive devices [15–17]. Therefore, we tested three bands with different levels of stiffness to exert different levels of assistive force on the participants. The design parameters of these bands were selected considering the suit deformation.

The estimated decrease in the maximum flexion moment during running at 2.5 m/s was used as the index for the assistance levels of the band modules. The participants tested band modules with different stiffness values, and a band module estimated to reduce the maximum flexion moment by 6.5% was set to correspond to the highest stiffness. This selection was based on feedback from a pilot study that indicated that bands stronger than this threshold induced discomfort during running. In addition to this band, bands expected to reduce the maximum flexion moment by 5.5% and 4.5% were selected as the test bands.

## Experimental setup

### Test protocol

To evaluate the assistive performance of the fabricated suit, tests were conducted with eight healthy male participants (age: 24.4 ± 3.8 y; height: 1.72 ± 0.05 m; weight: 74.5 ± 6.1 kg, mean ± standard deviation). Individuals who were sufficiently healthy to undergo the test protocol without any difficulties were selected as the participants. The specific exclusion and inclusion criteria for recruiting the participants are presented in the supplementary material. The experimental protocol was approved by the Chung-ang University Institutional Review Board, and the participants provided informed consent. Before the actual protocol, the participants underwent one day of training and at least two days of recovery to eliminate the effects of fatigue. Before starting the protocol, the standing metabolic rate for each participant was measured and later subtracted from the metabolic rate in each running condition to determine the energy expenditure caused only by the running motions. The protocol consisted of the following procedures: warmup running for 5 min followed by no-suit, Band 1, Band 2, Band 3, and no-suit conditions. To minimize the order effect, the no-suit condition was tested twice as the first and last step of the protocol. We used the average data from the two trials except for the additional analysis conducted to reduce the post hoc selection effect. The additional analysis was done using the condition that resulted in lower metabolic rates between the two no-suit conditions. The participants ran for 5 min at 2.5 m/s for each condition and took a break of at least 10 min between each consecutive run. The break duration was the same for all participants.

The order of band conditions for each participant was randomized by using a Latin square design, which is a design method for choosing the sequences of experimental protocols that can minimize the error from the order effect. To familiarize participants with running with the exosuit, the training session was conducted in a manner similar to that of the actual protocol, albeit with the use of a motion capture system.

### Data collection and analysis

**Fig. 5:**
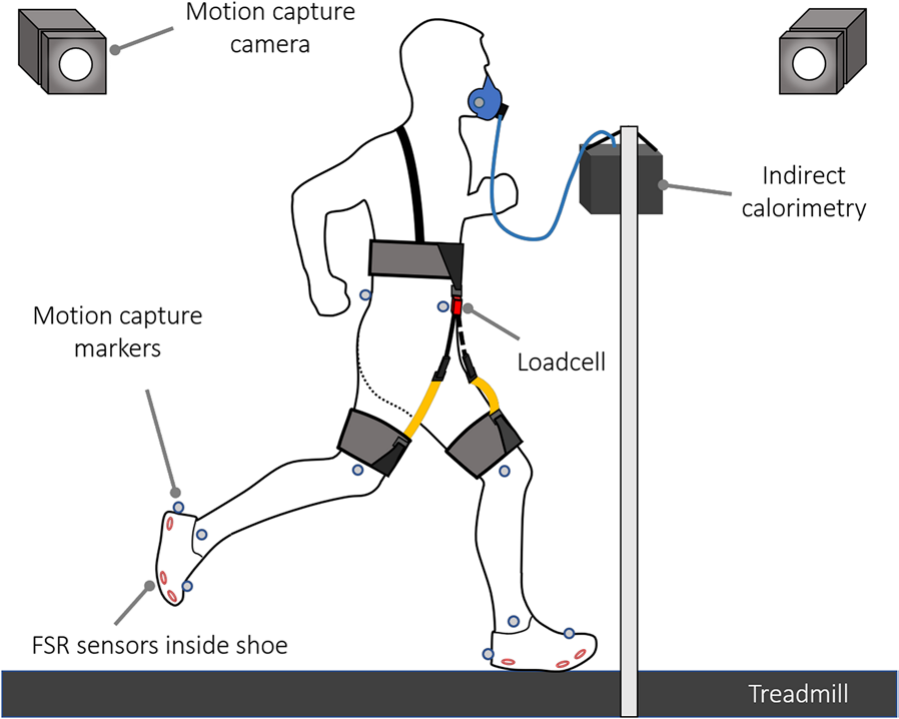
Experimental setup

Data were collected using the experimental setup shown in Fig. 5. An indirect calorimetry device (K5, COSMED, Italy) was used to obtain the VO2 and VCO2 data. These data were input to MATLAB (MathWorks, Natick, MA, USA) to calculate the metabolic rate for each condition by using the Brockway equation [18]. Only the data from the last 2 min of the 5-min run for each condition were used to obtain the steady-state response. All the metabolic rates were normalized by the body weight without considering the excess weight of the device. Six sets of motion capture cameras (T-10, Vicon, UK) were used to obtain the marker positions, which were then used to obtain the hip angle data. The motion capture markers were placed below the waist by a trained operator according to the Helen Hayes marker set. A loadcell (LSB205, FUTEK, USA) was attached to the right leg band module to measure the force from the band, and FSR sensors (FSR sensor contact, 0.5 in round, used with Trigno 4-Channel FSR adapter, Delsys, USA) were placed inside the shoes to detect ground contact. Data from the loadcell, FSR sensors, and motion capture system were collected and synchronized using data capture software (Vicon NEXUS, Vicon) before being processed with motion analysis software (Visual3D, C-Motion, USA).

Data from the loadcell were filtered using a low-pass filter with a cutoff frequency of 20 Hz. The cutoff frequency was selected considering the fact that the frequency of human activity is generally below 20 Hz, as reported by Khusainov et al. [19]. The FSR sensor data were processed using the method reported by Harle et al. [20]. Specifically, a median filter was used to reduce the noise, and the amplitude of the sensor signals was normalized. The data from a sensor were divided by the recorded maximum input from that sensor to ensure that all the data ranged from 0 to 1. When this method was applied to the data, the noise level did not exceed 0.1 when the sensors were in an unpressed, neutral state. Therefore, we adopted a threshold of 0.2 to distinguish sensor activation, which was used to identify foot contact. Using the sensor data, the stance and swing phase durations and duty rate of two phases in one GC were calculated.

Although a band with a higher stiffness was expected to exert a higher assistive force, the actual forces exerted by the bands did not always match this expectation. In tests for three of the eight participants, the highest force profiles were obtained while running with a band of medium stiffness. This phenomenon likely occurred because the force exerted by a band is related to not only the band stiffness but also the angle of the hip. Notably, the running motions of participants may have varied under different conditions, and the lower range of motion of the hip in the direction of extension could have resulted in less elongation of the band compared to that in the other conditions. Thus, the three test conditions were sorted in the order of the measured exerted forces rather than the band stiffness. These conditions were expressed as LOW, MED, and HIGH in ascending order of the maximum measured band force.

Owing to technical issues, the FSR sensor and motion capture data could not be acquired from participant P8, and thus, only the metabolic cost data for P8 were analyzed. For the FSR sensor and motion capture data, only the data from participants P1–P7 were analyzed.

### Statistics

We organized the data and conducted statistical analyses in MATLAB. The results pertaining to the metabolic cost and spatiotemporal parameters are reported as the mean ± standard error of the mean (s.e.m.). We performed a two-sided paired t-test to assess the difference in the metabolic rate and spatiotemporal parameters (stance duration, swing duration, stance phase, swing phase, stride frequency, and stride length) between the no-suit and BEST conditions (in which the participants exhibited the lowest metabolic rates). Furthermore, the effects of the different conditions (LOW, MED, HIGH, and no-suit) on the metabolic rates and spatiotemporal parameters were investigated through a one-way repeated measures analysis of variance (ANOVA). As a post-hoc test, we performed the two-sided paired t-test to compare the suit conditions to the no-suit condition to identify the significant changes. For all analyses, the significance level was set at p < 0.05. For all statistical tests relying on the normality assumption, the normality of the data was verified through the Jarque–Bera test. The normality assumption was met for all the data tested in this study.

## Results

### Metabolic cost

**Fig. 6:**
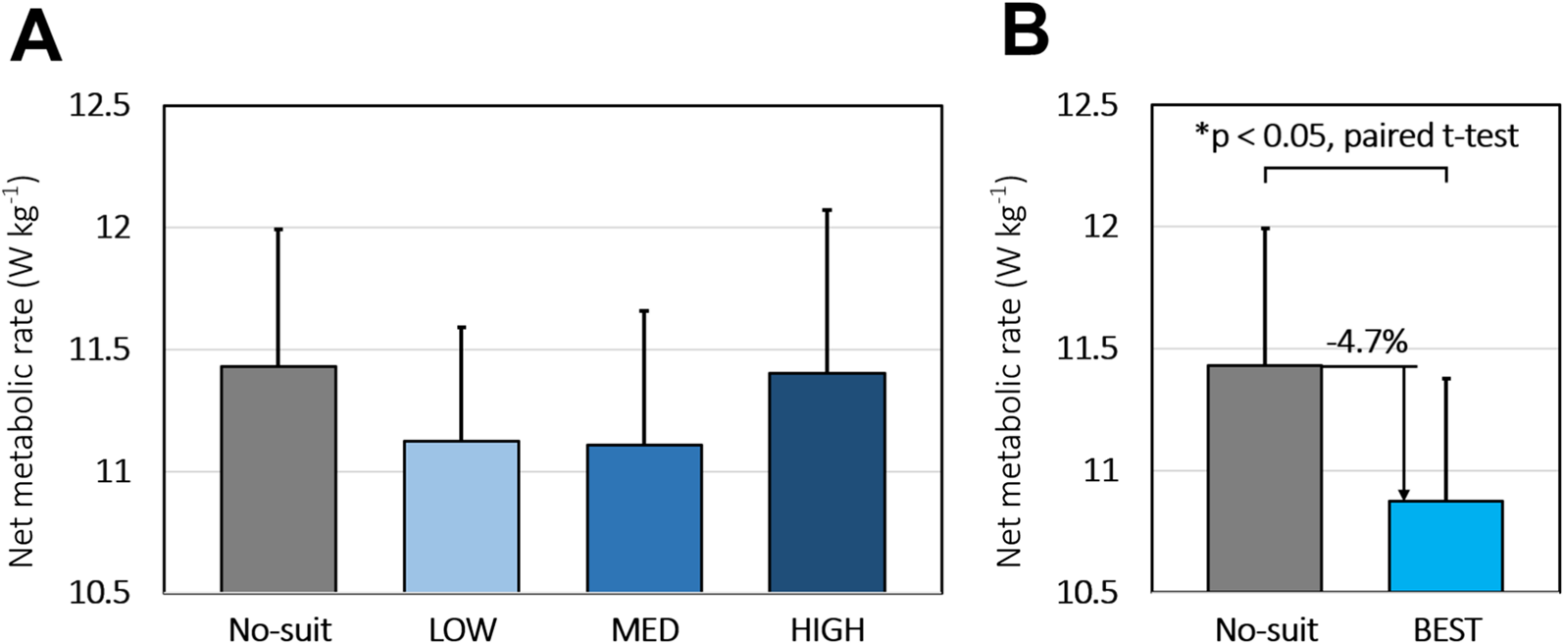
Metabolic cost results. The error bar shows the s.e.m. **A** Average metabolic rate of all participants across different conditions. A U shape can be observed in the results as the assistive force increases from the no-suit condition to the HIGH condition. **B** Average metabolic rate of BEST condition (best fitting band conditions for each participant, light blue bar) compared to average metabolic rate in no-suit condition (gray bar)

The metabolic rates during standing were found to have an average value of 2.05 ± 0.13 W/kg (mean ± s.e.m.). As the assistive force magnitude increased from the no-suit to the HIGH conditions, the average metabolic rate from all the participants exhibited a U shape, as shown in Fig. 6A. A similar U-shaped result was reported by Collins et al., who tested a wearable device for walking by varying the stiffness of the elastic component (mixed model, three-factor ANOVA, p < 0.05) [21]. However, the statistical analysis showed that the metabolic rates did not significantly vary across conditions (one-way repeated measures ANOVA, p > 0.05).

Considering the condition with the lowest metabolic cost measured as the best-fitting band condition for each participant (referred to as BEST), the metabolic rate in these conditions was reduced by an average of −4.7 ± 1.4% (mean ± s.e.m., p = 0.017, two-sided paired t-test) compared to the average of the two no-suit conditions. The metabolic rate reductions in the best resulting conditions for each participant are presented in the supplementary material.

However, the method of choosing a condition that resulted in the lowest metabolic cost among the three band conditions and comparing it with the average of two no-suit conditions can be subject to errors from post hoc selection. The metabolic cost data has variability due to noise, and because we chose the data with the best results from three conditions, the reductions may be a result of noise from metabolic data rather than the effect of the suit. To reduce this potential post hoc selection effect, we compared the metabolic rates of the BEST condition to those of the condition that showed the lower metabolic rate between the two no-suit conditions. Statistically significant results were obtained with an average reduction of − 2.5 ± 1.1% (mean ± s.e.m., p < 0.05, two-sided paired t-test).

The condition of best fit for the largest number of participants (four) was the MED condition. The LOW and HIGH conditions were the best conditions for two participants each. Among the eight participants, six of the participants exhibited a reduced metabolic cost while running with the suit in at least one of the three conditions compared to the average of no-suit conditions. One participant exhibited a negligible difference in the best resulting band condition, and another participant exhibited an increase in the metabolic cost while wearing the suit in all three conditions. The metabolic rate values measured for all participants are listed in Table 1.

**Table 1:** Metabolic power measured from the participants for each test condition and best condition where the participants displayed lowest metabolic rates

Participant	No-suit (W/kg)	Low (W/kg)	Med (W/kg)	High (W/kg)	Best (W/kg)
P 1	9.27	9.52	8.64	8.95	8.64
P 2	12.62	12.54	12.21	12.95	12.21
P 3	12.51	12.63	12.51	13.45	12.51
P 4	12.36	11.56	12.95	11.86	11.56
P 5	9.54	9.57	9.70	9.64	9.57
P 6	13.39	12.26	12.00	13.85	12.00
P 7	9.99	9.94	9.91	9.78	9.78
P 8	11.76	10.97	10.95	10.73	10.73

### Level of assistive force

**Fig. 7:**
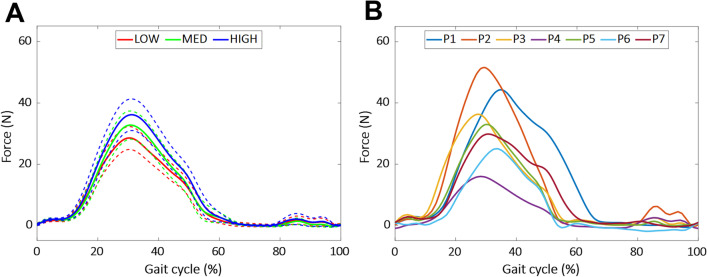
Force profiles exerted by bands during GC. **A** Average band force profiles in Low, Med, and High conditions. The dotted line indicates the s.e.m. **B** Average band force profiles for participants P1–7 in conditions with highest metabolic rate reductions

Data from the loadcell, which measured the actual pulling forces from the bands, were acquired for seven of eight participants. The data showed that the assistance levels of the three bands did not match the expectation of bands with higher stiffness values, resulting in higher assistance force profiles. For three participants, the highest maximum assistive forces were attained with the band of medium stiffness instead of the band of the highest stiffness. As mentioned previously, the bands were rearranged in order of actual peak forces. The average forces exerted by the bands in the LOW, MED, and HIGH conditions, according to the GC, are shown in Fig. 7A. The band forces during gait for the seven participants with individual best-fitting bands are shown in Fig. 7B.

### Spatiotemporal parameters

Using the FSR sensor data, the timings of heel-strike and toe-off events were identified for the seven participants. Table 2 lists the average stance and swing phase durations and duty rates of the two phases, along with the step frequencies and step length under each condition.

**Table 2:** Spatiotemporal parameters for each test condition and BEST condition (with lowest metabolic rates) (mean ± s.e.m.)

Parameter	No-suit	Low	Med	High	Best
Stance duration (ms)	315.38 ± 6.85	315.53 ± 7.17	325.40 ± 7.44	325.90 ± 8.25	324.64 ± 7.60
Swing duration (ms)	404.04 ± 11.50	394.77 ± 10.08	403.97 ± 11.32	397.11 ± 12.47	403.12 ± 13.23
Stance phase (%)	43.9 ± 0.9	44.4 ± 0.8	44.7 ± 1.1	45.1 ± 1.2	44.7 ± 1.2
Swing phase (%)	56.1 ± 0.9	55.6 ± 0.8	55.4 ± 1.1	54.9 ± 1.2	55.3 ± 1.2
Stride frequency (Hz)	1.392 ± 0.027	1.410 ± 0.024	1.373 ± 0.019	1.385 ± 0.023	1.376 ± 0.023
Stride length (m)	1.799 ± 0.033	1.776 ± 0.031	1.823 ± 0.025	1.808 ± 0.031	1.819 ± 0.030

There was a significant change in the spatiotemporal parameters in the conditions (LOW, MED, HIGH, and no-suit) for the stance duration (one-way repeated measures ANOVA, p < 0.01). The stance duration increased from 315.38 ms in the no-suit condition to 325.40 and 325.90 ms in the MED and HIGH conditions, corresponding to an increase of 3.2% (two-sided paired t-test, p < 0.05) and 3.3% (two-sided paired t-test, p = 0.065), respectively. The other spatiotemporal parameters did not change significantly across the conditions (one-way repeated measures ANOVA, all p > 0.05).

Furthermore, the average stance duration showed a trend toward increase from 315.38 ms in the no-suit condition to 324.64 ms in the BEST condition, corresponding to an increase of 2.9% (two-sided paired t-test, p = 0.062). Although the average stance duration increased slightly, the average swing duration did not change considerably, decreasing by 0.2% from 404.04 ms in the no-suit condition to 403.12 ms in the BEST condition. Thus, the average duty rate of the stance phase increased from 43.9% in the no-suit condition to 44.7% in the BEST condition. Although changes in the spatiotemporal parameters of the BEST conditions were observed for the individuals, none of them was statistically significant (two-sided paired t-test, all p > 0.05).

## Discussion

We developed a lightweight and low-profile device capable of enhancing human running economy. The fabricated passive suit weighs 609 g, less than the existing mobile devices developed for assistive running that target the hip joint (Table 3). Moreover, the suit has a small extrusion of 2.5 cm from the body in the standing posture and is comfortable to wear for long periods owing to its low profile.

**Table 3:** Specifications of existing assistive devices for running, in order of appearance

Study	Target joint	Portable/Tethered	Device type	Device mass (kg)	Comparison performed	Metabolic reduction (%)
Lee et al. [5]	Hip	Tethered	Active	0.81	Powered vs. No_Exo^a^	8
Witte et al. [15]	Ankle	Tethered	Active	0.88	Powered vs. No_Exo	14.6
Nasiri et al. [8]	Hip	Portable	Passive	1.8	Device on vs. No_Exo	8
Simpson et al. [9]	Lower extremity^b^	Portable	Passive	0.05	Device on vs. No_Exo	6.4
Kim et al. [4]	Hip	Portable	Active	5	Powered vs. No_Exo	4
Yang et al. (This study)	Hip	Portable	Passive	0.61	Device on vs. No_Exo	4.7

No_Exo^a^ condition performed by removing the effective mass of the device from the carried load

Lower extremity^b^ indicates that the target joint is unspecified. The device is attached to the two ankles, and thus, the entire lower extremity is affected

Evaluation of the suit on eight participants confirmed that wearing the suit with proper bands could decrease the metabolic energy expended during running compared to that incurred when running without the device. The level of assistive force from the suit and spatiotemporal parameters were analyzed for seven participants. Although the principle behind the suit design was to relieve the biological hip flexion torque over a specific range of motion, it could not be definitively concluded that the assistive force was the only reason for the reduced metabolic rate. Notably, other aspects such as the change in spatiotemporal parameters, for example, the optimal running frequency, can also affect the metabolic cost of running [9].

The passive suit influenced the stance duration considerably. In particular, in the MED condition, the stance duration was 3.2% shorter than that in the no-suit condition. Six of the eight participants exhibited metabolic reductions in the MED condition. Although not statistically significant, the stance duration in the BEST condition was 2.9% longer than that in the no-suit condition. The elongated stance duration could have been a direct cause of changes in the metabolic rate. Arellano et al. estimated that the metabolic power used for bodyweight support and forward propulsion comprises up to 80% of the total metabolic cost of running [22]. Because the bodyweight support and forward propulsion occur in the stance phase of the leg, longer stance durations suggest lower levels of maximum activation in the muscles if the total impulse from the foot to the ground is identical for each step, which likely reduces the metabolic rate. The effects of this change on the spatiotemporal parameters should be further investigated.

Theoretically, the exosuit did not require any additional extension torque from the hip when the bands were stretched because the stretching occurred when the hip flexion torque was needed for running. However, the running kinetics and kinematics may differ between individuals. For certain individuals, the hip angle at which the hip torque changes from extension to flexion torque may not correspond to a flexion angle of 18°, as assumed in this study. Users who do not satisfy this assumption will likely require additional biological extension moments to stretch the bands. These moments will act as a metabolic burden and result in the nonideal performance of the exosuit. In this scenario, the HIGH condition with stronger assistive force will require additional activation of the hip extensors and will therefore not be the best condition to increase the metabolic efficiency.

Another interesting finding was that bands with higher stiffness values did not always exert greater force profiles on the participants. Three of the participants, P2, P6, and P7, exhibited higher force profiles when using the medium stiffness band than when using the band with the highest stiffness. This unexpected outcome can likely be attributed to the reduction in the hip range of motion while using the band of a higher stiffness. For example, the maximum extension of P2 was 2.2° lower when using the band of the highest stiffness than that when using the band of medium stiffness. Because the angle at the maximum extension is directly correlated with the maximum force of the band, the unexpected order of the band force profiles can likely be explained by this difference in the range of motion.

The proposed suit was lightweight primarily because elastic bands were used to generate the assistive force profiles. The potential of using elastic bands in wearable devices has been demonstrated in previous studies. The exotendon, composed of an elastic band attached to the shoe, attained a metabolic reduction of 6.4% while running at 2.7 m/s. In addition to the fact that the exotendon has a different working principle than that of the proposed device, one of the reasons for such a high reduction is the extremely low weight of the device (50 g). The reduction can also be attributed to the fact that the device is stably anchored to the body, as it is directly attached to the shoe. Stable anchoring can help deliver more force to the body without the dissipation of energy from the deformation of the body. Notably, although the exotendon achieves a larger reduction than the proposed device, it involves certain limitations such as possibilities of getting caught in obstacles and limited usage on irregular terrain due to the form factor of the device.

Our proposed device has a slim design that has minimal extrusion from the body. Designs like this are particularly desirable for wearable devices because devices that do not alter the outline of the human body can be worn underneath everyday clothing. This aspect is a valuable characteristic of the device, as several individuals may wish to conceal their use of an assistive device. According to Jung et al., most elderly individuals preferred exoskeletons to be as inconspicuous as possible because most of them did not want to advertise their need for assistive devices [23].

The assistive mechanism of the device developed by Panizzolo et al. is similar to that of the proposed device, with the major difference pertaining to the target motion of the device [12]. Notably, the former device aims to assist the elderly. If walking assistance via supporting hip flexion can be applied to all individuals, it may be possible to use a single slim suit-type wearable device to help with both walking and running. Future work could evaluate the proposed passive suit in diverse gait conditions including walking.

## Conclusions

This paper presented a passive exosuit that can reduce the metabolic energy cost required for running. The fabricated device has a low-profile design and an extremely low weight of 609 g. The assistive potential of the fabricated exosuit was tested via human experiments involving eight participants. The results showed that running with the appropriate bands for the individual’s size could reduce the metabolic rate during running by − 4.7 ± 1.4% (p = 0.017) compared to that incurred while running without the suit. According to Teunissen et al., a metabolic reduction of 19% can be achieved by reducing the body weight by 25% [24]. Assuming linear correlations between the reduced body weight and metabolic cost reduction, the metabolic reduction of 4.7% achieved using the exosuit is equivalent to reducing the body weight of the runner by 6.45%.

This metabolic reduction of 4.7% is a competitive result compared to the state-of-the-art results achieved using the existing exoskeletons for assistive running (Table 3). Although the reduction rate alone may seem exiguous compared to those reported previously, the proposed device is promising because of its low weight. In particular, the proposed exosuit has the lowest weight among the considered portable devices that target the hip joint. The weight of the proposed exosuit is only 33.8% and 12.2% of those of the devices proposed by Nasiri et al. and Kim et al., respectively [4, 8].

When using a wearable device, the optimal assistance levels may vary between individuals [15–17]. To test the capabilities of the suit under conditions involving an individual’s unknown optimal assistance level, we conducted experiments under the best resulting conditions from three test conditions, similar to those in the research of Pannizzolo and Barazesh [12, 25]. In future work, we plan to conduct additional experiments to further verify the results of the best resulting conditions.

## Supplementary Information

**Additional file 1: Figure S1.** Weight breakdown of device. Participant inclusion/exclusion criteria.

**Additional file 2: Figure S2.** Graph showing metabolic cost results for all conditions. Video showing working mechanism of flexion passive exosuit. The elongation of the band according to the hip extension during the running gait is shown.

## Data Availability

All data generated or analyzed during this study are included in this article.

## Abbreviations

GC: Gait cycle
s.e.m.: Standard error of the mean

## References

[1] Williams KR, Cavanagh PR (1987). Relationship between distance running mechanics, running economy, and performance. J Appl Physiol.

[2] Hoogkamer W, Kipp S, Spiering BA, Kram R (2016). Altered running economy directly translates to altered distance-running performance. Med Sci Sports Exerc.

[3] Sawicki GS, Beck ON, Kang I, Young AJ (2020). The exoskeleton expansion: improving walking and running economy. J Neuroeng Rehabil.

[4] Kim J, Lee G, Heimgartner R, Revi DA, Karavas N, Nathanson D (2019). Reducing the metabolic rate of walking and running with a versatile, portable exosuit. Science.

[5] Lee G, Kim J, Panizzolo FA, Zhou YM, Baker LM, Galiana I (2017). Reducing the metabolic cost of running with a tethered soft exosuit. Sci Robot.

[6] Elliott G, Sawicki GS, Marecki A, Herr H. The biomechanics and energetics of human running using an elastic knee exoskeleton. In 2013 IEEE 13th Int Conf Rehabil Robot (ICORR). 2013;1–6. 10.1109/ICORR.2013.6650418 24187237

[7] Cherry MS, Kota S, Young A, Ferris DP (2016). Running with an elastic lower limb exoskeleton. J Appl Biomech.

[8] Nasiri R, Ahmadi A, Ahmadabadi MN (2018). Reducing the energy cost of human running using an unpowered exoskeleton. IEEE Trans Neural Syst Rehab Eng.

[9] Simpson CS, Welker CG, Uhlrich SD, Sketch SM, Jackson RW, Delp SL (2019). Connecting the legs with a spring improves human running economy. J Exp Biol.

[10] Silder A, Whittington B, Heiderscheit B, Thelen DG (2007). Identification of passive elastic joint moment–angle relationships in the lower extremity. J Biomech.

[11] Novacheck TF (1998). The biomechanics of running. Gait Posture.

[12] Panizzolo FA, Bolgiani C, Di Liddo L, Annese E, Marcolin G (2019). Reducing the energy cost of walking in older adults using a passive hip flexion device. J Neuroeng Rehabil.

[13] Wei T, Chen W, Wu S, Zhou T, Xiong C. The compensatory strategies among lower limb muscles and its effect on metabolic cost when hip assisted by passive elastic exotendon. In 2019 IEEE 4th Int Conf Adv Robot Mechatron. 2019;593–8.

[14] Lee G, Ding Y, Bujanda IG, Karavas N, Zhou YM, Walsh CJ. Improved assistive profile tracking of soft exosuits for walking and jogging with off-board actuation. In 2017 IEEE/RSJ Int Conf Intel Robot Syst. 2017;1699–1706.

[15] Witte KA, Fiers P, Sheets-Singer AL, Collins SH (2020). Improving the energy economy of human running with powered and unpowered ankle exoskeleton assistance. Sci Robot.

[16] Zhang J, Fiers P, Witte KA, Jackson RW, Poggensee KL, Atkeson CG (2017). Human-in-the-loop optimization of exoskeleton assistance during walking. Science.

[17] Ding Y, Kim M, Kuindersma S, Walsh CJ (2018). Human-in-the-loop optimization of hip assistance with a soft exosuit during walking. Sci Robot.

[18] Brockway JM (1987). Derivation of formulae used to calculate energy expenditure in man. Hum Nutr Clin Nutr.

[19] Khusainov R, Azzi D, Achumba IE, Bersch SD (2013). Real-time human ambulation, activity, and physiological monitoring: taxonomy of issues, techniques, applications, challenges and limitations. Sensors.

[20] Harle R, Taherian S, Pias M, Coulouris G, Hopper A, Cameron J (2012). Towards real-time profiling of sprints using wearable pressure sensors. Comput Commun.

[21] Collins SH, Wiggin MB, Sawicki GS (2015). Reducing the energy cost of human walking using an unpowered exoskeleton. Nature.

[22] Arellano CJ, Kram R (2014). Partitioning the metabolic cost of human running: a task-by-task approach. Integr Comp Biol.

[23] Jung MM, Ludden GD (2019). What do older adults and clinicians think about traditional mobility aids and exoskeleton technology?. ACM Trans Hum Robot Interact.

[24] Teunissen LP, Grabowski A, Kram R (2007). Effects of independently altering body weight and body mass on the metabolic cost of running. J Exp Biol.

[25] Barazesh H, Sharbafi MA (2020). A biarticular passive exosuit to support balance control can reduce metabolic cost of walking. Bioinspir Biomim.

