# Supplementary material for: Reducing the energy cost of running using a lightweight, low-profile elastic exosuit
Source: J Neuroeng Rehabil. 2021 Aug 30;18:129. doi: 10.1186/s12984-021-00928-x (PMC8404320; doi:10.1186/s12984-021-00928-x)
Supplement: Supplementary file 1 — Additional file 1: Figure S1. Weight breakdown of device. Participant inclusion/exclusion criteria. [file 12984_2021_928_MOESM1_ESM.pdf]

Supplementary information

Device weight breakdown

The mass of each device component is shown in Fig. S1.

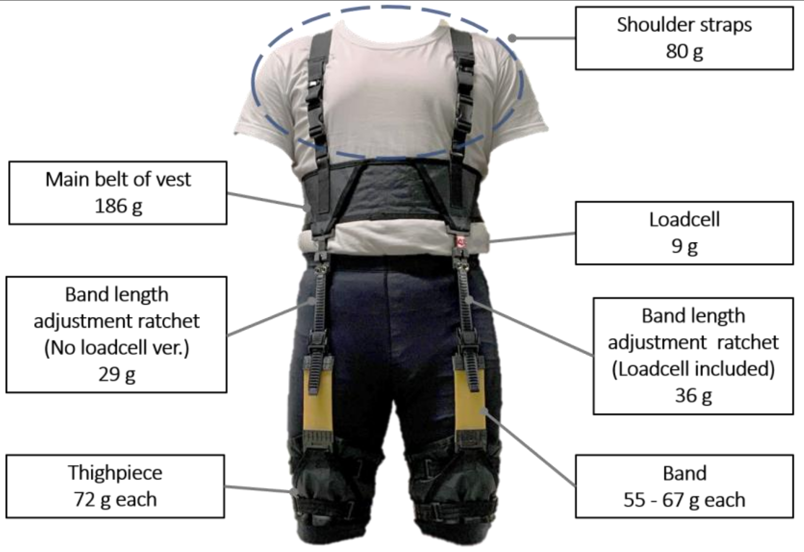

Fig. S1 Weight breakdown of proposed device. The weight of the ratchet system for band adjustment depends on whether the loadcell is connected. Only the ratchet on the right side is connected with the loadcell during experiments. The weight of the band module depends on the band stiffness. The suit weight, 609 g, is based on the heaviest band.

Participant inclusion/exclusion criteria

When recruiting participants that could effectively undergo the protocol, we used the following inclusion/exclusion criteria:

1. Inclusion criteria

1.1. Age / sex: Adult male/female (aged 20–50 y)

1.2 The person must be responsive and not exhibit any communication difficulties to ensure that s/he can understand the test process.

1.3 The person must be healthy to ensure that his/her physical capabilities can be evaluated.

1.4 The person must not have suffered any musculoskeletal injuries in the past year.

1.5 The person must provide written consent.

2. Exclusion criteria

2.1. The person has an ongoing musculoskeletal illness.

2.2. The person has observable gait abnormalities when walking 10 m.

2.3. The person has a record of musculoskeletal illness, such as bone fracture or ligament tear.

2.4. The person has an ongoing illness related to the neurological/cardiovascular systems.

2.5. The person has observable abnormalities when performing object manipulation.

Individual reduction graphs

Figure S2 shows the metabolic rate bar graph of each participant in all test conditions.

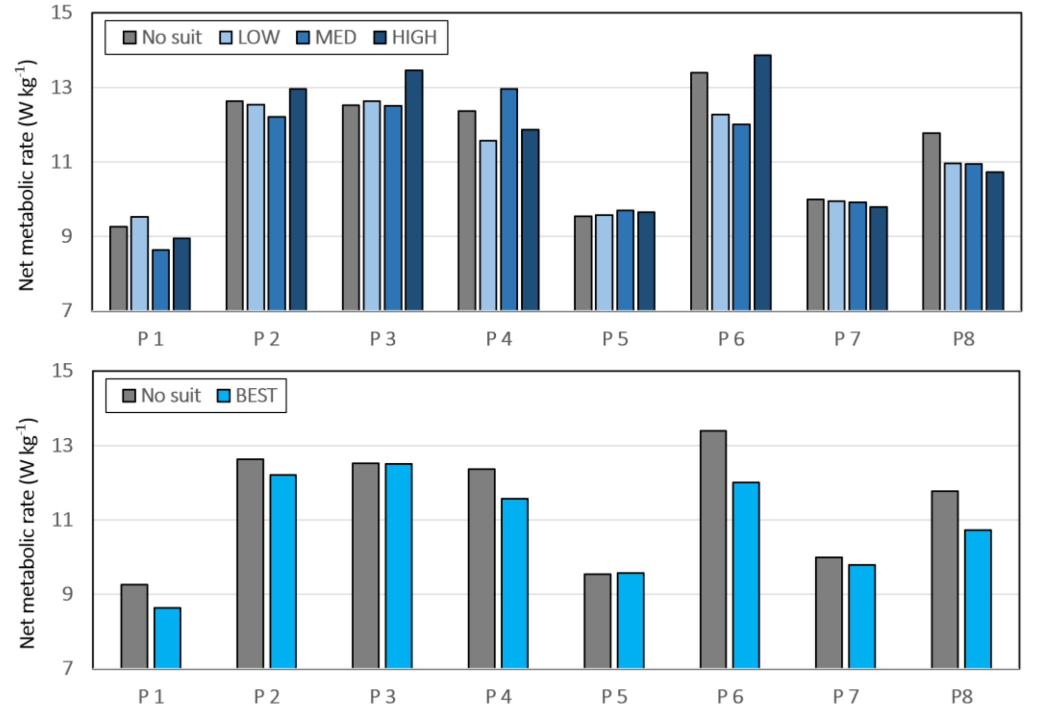

Fig. S2 (Top) Bar graph showing metabolic cost results for all conditions (Bottom) Bar graph comparing metabolic cost of no-suit and best conditions for individuals.
